# The burden of tuberculosis among adolescents and young adults in five Asian countries from 1990 to 2019

**DOI:** 10.1186/s13690-023-01160-w

**Published:** 2023-08-08

**Authors:** Yu Siyu, Li Shihong, Liu Hanzhao, Xu Qiufang, Liu Jingyi, Cai Fengzhu, Xiao Shaotan, He Gengsheng

**Affiliations:** 1 School of Public Health/Key Laboratory of Public Health Safety, Ministry of Education, Department of Nutrition and Food Science, Fudan University, Shanghai, 200032 China; 2 Shanghai Pudong New Area Center for Disease Control and Prevention, Fudan University Pudong Institute of Preventive Medicine, Shanghai, 200136 China; 3 Shanghai Qingpu Area Center for Disease Control and Prevention, Shanghai, 201799 China

**Keywords:** Tuberculosis, Incidence, Mortality, Trends, Asia

## Abstract

**Background:**

Previous studies have shown that the risk of tuberculosis (TB) increases dramatically during adolescence. The objective of this article was to analyze the burdens and trends of TB incidence and mortality rates in Asian adolescents and young adults.

**Methods:**

Time series ecological study of TB incidence and mortality rates of adolescents and young adults aged 10–24 years from 1990 to 2019, using data extracted from the Global Burden of Disease website for 5 Asian countries. The annual percentage change was calculated by joinpoint regression analysis to estimate the trends in the age-standardized incidence rate (ASIR) and age-standardized death rate (ASDR).

**Results:**

The highest ASIR per 100,000 person-years in 2019 was in Mongolia [74 (95% uncertainty interval (UI), 51 to 105)], while the lowest was in Japan [4 (95% UI, 2 to 6)]. The highest ASDR per 100,000 person-years was in Mongolia [2 (95% UI, 1 to 3)], while the lowest was in Japan [0.009 (95% UI, 0.008 to 0.010)]. As the absolute number of cases and deaths decreased from 1990 to 2019, the ASIRs and ASDRs in all five countries also decreased.

**Conclusions:**

Our finding revealed that although all five countries in Asia experienced descending TB incidence and mortality trend in past three decades, the trends were especially significant in developed countries and varied across geographic regions. This study may be crucial in helping policymakers make decisions and allocate appropriate resources to adolescent TB control strategies.

**Table:** 

**Text box 1. Contributions to the literature**
• With the continuous attention and increasing investment of resources on public health, the morbidity and mortality of adolescents with TB have shown an overall downward trend in Asian, while fluctuated briefly with the adjustment of public health strategies in some periods.
• Given the incomplete reporting systems in some countries, a certain proportion of adolescent patients are not diagnosed or formally reported, which may affect the formulation of health policies.
• These findings contribute to recognized gaps in the literature, including the importance of rationalizing public health resources, improving screening, reporting systems, and studies for TB prevention interventions in adolescents.

## Introduction

Adolescence is recognized as a pivotal period of development that lays the foundation for future health and well-being [1, 2]. Historical data suggest that in individuals between the ages of 12 and 24 years, there may be an increase in the risk of progression to disease after infection compared with children or older adults [3]. However, TB in adolescence is an overlooked epidemic with a staggering disease burden during a long historical period [4], and a considerable proportion of adolescents face age-related health services challenges in accessing appropriate health care when transitioning from childhood to adulthood, particularly in tuberculosis-endemic regions where special adolescent health services are usually inadequate [2]. This situation has led the World Health Organization (WHO) to place increasing emphasis on improving the estimates of the burden of morbidity and mortality from TB in young people and its contribution to the global burden of TB [5].

Mycobacterium tuberculosis causes active tuberculosis disease in 10 million people annually [6], with nearly 50% diagnosed in Asian countries in 2019. Since 2000, even though the prevalence rate of TB has declined significantly in different Asian regions, as well as the mortality rate [7], there are still seven countries on the Asian continent that are included in the top 22 countries with a high TB burden [8]. The epidemiology of TB shows substantial geographic heterogeneity, and its incidence varies widely between high- and low-risk Asian countries [9]. Efforts to explore whether TB elimination strategies neglect adolescents and young adults aged 10–24 years in Asian regions and estimate the burden of TB among them in Asia remain a formidable challenge [10, 11]. However, the latest data on the trends in the incidence and death rates of young TB patients and advanced statistical modeling of TB burden trends remain poorly understood in some Asian countries [12]. Therefore, in this study, we designed the present study to evaluate the burden and trend of TB ASIR, ASDR and develop effective strategies for the prevention of TB and to extend the life expectancy of adolescent and young adult (10–24 years old) in five Asian countries, including Mongolia, China, Singapore, the Republic of Korea, and Japan, from 1990 to 2019 in relation to year and sex, based on the GBD 2019 study.

## Materials and methods

### Data sources

A time series ecological study was conducted which considered all incidence and deaths by tuberculosis that occurred among adolescents and young adults aged 10–24 years from 1990 to 2019 in five Asian countries. The data sources and statistical methods of the GBD 2019 have been described in other studies [13, 14]. In brief, in the GBD study, data were estimated for 369 diseases and injuries at global and regional levels from 1990 to 2019. We extracted data from the Global Health Data Exchange (GHDx) website (http://ghdx.healthdata.org/gbd- results-tool). We used the term “tuberculosis” as the “cause” and the terms “incidence” and “death” as the “measure”. Data on the ASIR and ASDR at global and national levels were extracted from the GBD 2019 [15]. The geographic locations included China, Mongolia, the Republic of Korea, Singapore, and Japan. Estimates were reported with 95% uncertainty intervals (UIs), which were generated using data simulation bootstrapping techniques, generating a data set with 1000 iterations of each country.

## Estimates of the annual percentage change (APC)

The APC was estimated to test time trends in ASIRs and ASDRs from 1990 to 2019 using joinpoint regression analysis (Joinpoint Regression Program, Version 4.7.0.0, USA) [16]. We estimated the time trend by the APC with 95% confidence intervals (CIs) for each segment identified by the model and tested which trends between joinpoints were significantly different from zero. A *P* value < 0.05 was considered statistically significant. The ASIR or ASDR was considered to show an increasing trend if both the APC value and the lower 95% CI were > 0 and a decreasing trend if both the APC value and the upper 95% CI were < 0. Furthermore, an “increasing” trend was characterized as a significant APC > 0; and a “decreasing” trend was characterized as a significant APC < 0.

## Results

### Incidence and death among adolescents and young adults aged 10–24 years in 2019

The number of incident cases of TB among adolescents and young adults aged 10–24 years worldwide in 2019 was 1,816,495 (ASIR, 97 per 100,000 person-years), and the number of deaths was 58,340 (ASDR, 3 per 100,000 person-years). Estimates with uncertainty intervals for adolescents and young adults aged 10–24 years by 5 Asian countries were showen in Table 1. The ASIR per 100,000 person-years in 2019 was highest in Mongolia [74 (95% UI, 51 to 105)], followed by China [37 (95% UI, 27 to 49)], the Republic of Korea [19 (95% UI, 13 to 27)], and Singapore [16 (95% UI, 10 to 22)], and lowest in Japan [4 (95% UI, 2 to 6)] (Table 1). The ASDR per 100,000 person-years was also highest in Mongolia [2 (95% UI, 1 to 3)] (Table 1). There were 564 deaths in China (95% UI, 477 to 667), which accounted for 1% of the global deaths. Among adolescents and young adults aged 10–24 years worldwide and in Mongolia, the ASIRs were higher among females than males. The ASDRs were lower among females than males among adolescents and young adults worldwide and in Mongolia.

**Table 1:** Incidence and deaths with percentage changes of tuberculosis for both sexes among adolescents and young adults aged 10–24 years between 1990 and 2019

Location	1990		2019		Between 1990 and 2019	
	**Incident cases** **ASIR (95% UI)**	**Death cases** **ASDR (95% UI)**	**Incident cases** **ASIR (95% UI)**	**Death cases** **ASDR (95% UI)**	**Percentage change,** **ASIR (95% UI)**	**Percentage change,** **ASDR (95% UI)**
Global	2,232,379(3025089to1582383) 144(195to102)	111,785(124536to99950) 7(8to6)	1,816,495(2440144to1298492) 97(131to69)	58,340(65581to51857) 3(3to2)	-1.3 (-1.4to-1.2)	-3.1 (-3.2to-2.9)
China	289,202(380685to209122) 79(105to57)	7806(8963to6738) 2(2to1)	85,120(111864to62646) 37(49to27)	564(667to477) 0(0to0)	-2.3 (-2.5to-2.1)	-7.5 (-7.7to-7.3)
Singapore	289(409to190) 35(49to23)	2(2to1) 0(0to0)	120(171to81) 16(22to10)	0(0to0) 0(0to0)	-1.9 (-2.3to-1.4)	-7.6 (-7.9to-7.3)
Japan	2870(4189to1809) 10(14to6)	10(10to9) 0(0to0)	731(1064to474) 4(6to2)	1(1to1) 0(0to0)	-3.2 (-3.5to-2.8)	-5.2 (-5.4to-5)
Republic of Korea	10,445(13109to8017) 79(99to60)	325(360to291) 2(2to2)	1661(2338to1140) 19(27to13)	7(9to6) 0(0to0)	-4.2 (-4.6to-3.7)	-10.9 (-11.6to-10.3)
Mongolia	846(1094to613) 119(154to86)	39(51to29) 5(7to4)	547(768to378) 74(105to51)	20(29to14) 2(3to1)	-1.5 (-1.7to-1.4)	-2.8 (-3.3to-2.4)

*ASDR* Age-standardized death rate per 100,000 person-years, *ASIR* Age-standardized death rate per 100,000 person-years, *UI* Uncertainty interval

### Changes in tuberculosis incidence and death rates among adolescents and young adults aged 10–24 years between 1990 and 2019.

**Fig. 1:**
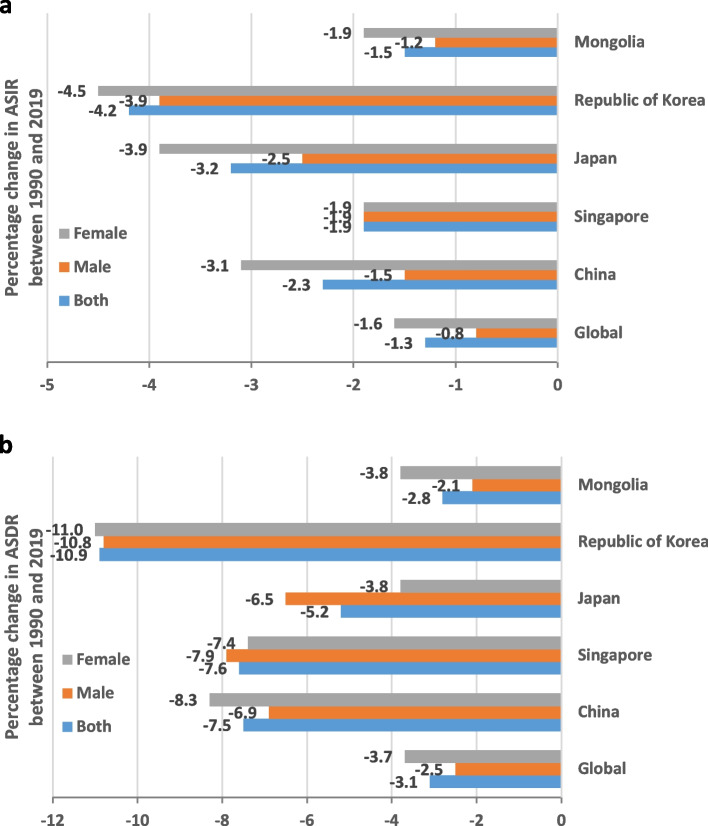
Percentage changes in the ASIR (**a**) and ASDR (**b**) of tuberculosis among adolescents and young adults aged 10–24 years worldwide and in five Asian countries between 1990 and 2019 according to sex. ASDR, age-standardized death rate. ASIR, age-standardized incidence rate

On analysis of TB trends among adolescents and young adults using Joinpoint regression, there were significant decreases in the TB incidence rates and death rates worldwide and in 5 Asian countries from 1990 to 2019. The global change in the ASIR between 1990 and 2019 was -1.3, and the change in the ASDR was -3.1 (Fig. 1a, b; Table 1). There were obvious decreases in the ASIR and ASDR between 1990 and 2019 in all five Asian countries (Fig. 1; Table 1). The greatest decrease in the ASIR was in the Republic of Korea [− 4.2 (95% UI, − 4.6 to − 3.7)], followed by Japan [− 3.2 (95% UI, − 3.5 to − 2.8)], China [− 2.3 (95% UI, − 2.5 to − 2.1)], Singapore [− 1.9 (95% UI, − 2.3 to − 1.4)], and Mongolia [− 1.5 (95% UI, − 1.7 to -1.4)] (Fig. 1a, Table 1). The greatest decrease in the ASDR was in the Republic of Korea [− 10.9 (95% UI, − 11.6 to − 10.3)], followed by Singapore [− 7.6 (95% UI, − 7.9 to − 7.3)], China [− 7.5 (95% UI, − 7.7 to − 7.3)], Japan [− 5.2 (95% UI, − 5.4 to − 5)] and Mongolia [− 2.8 (95% UI, − 3.3 to − 2.4)] (Fig. 1b, Table 1). Decreases in the ASIR were higher among females than males in all countries except Singapore. Decreases in the ASDR were higher among males than females in two countries: Japan and Singapore.

**Fig. 2:**
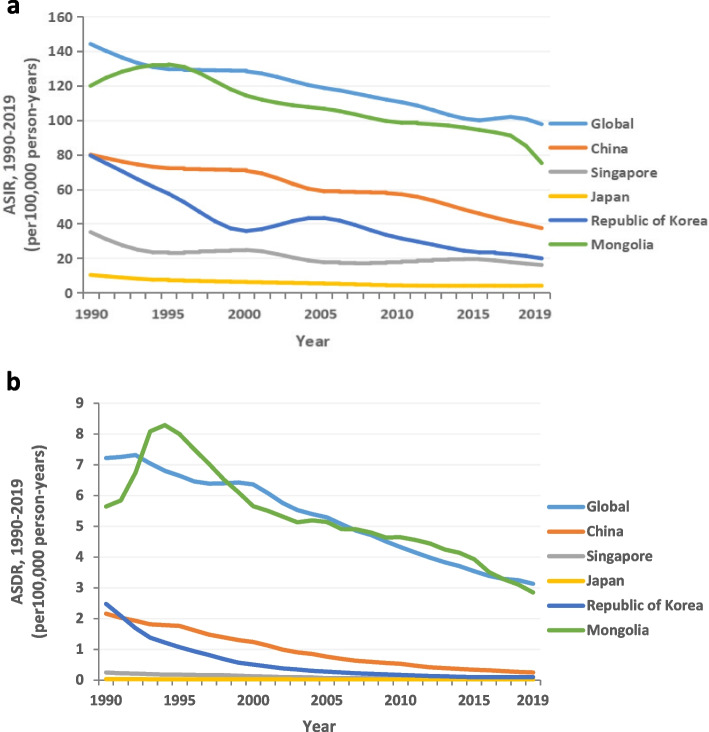
Time trends of the ASIRs (**a**) and ASDRs (**b**) of tuberculosis among adolescents and young adults aged 10–24 years worldwide and in five Asian countries from 1990 to 2019. ASDR, age-standardized death rate. ASIR, age-standardized incidence rate

The greatest decrease in the ASIR per 100,000 person-years was observed in the Republic of Korea, from 79 (95% UI, 60 to 99) in 1990 to 19 (95% UI, 13 to 27) in 2019 (Fig. 2a, Table 1). Mongolia initially showed an increasing trend in the ASIR from 1990 to 1995, but this then decreased continuously down to 74 (95% UI, 51 to 105) per 100,000 person-years in 2019. The ASIR in the Republic of Korea initially decreased from 1990 to 2000, then increased and peaked in 2005, and subsequently decreased continuously to 19 (95% UI, 13 to 27) per 100,000 person-years in 2019. In China, the ASIR showed a relatively gentle decline from 79 (95% UI, 57 to 105) to 37 (95% UI, 27 to 49) per 100,000 person-years from 1990 to 2019 (Fig. 2a, Table 1). The ASIR in Japan showed a slight downwards trend from 1990 to 2019. The greatest decrease in the ASDR per 100,000 person-years was observed in the Republic of Korea, from 2 (95% UI, 0 to 2) in 1990 to 0 (95% UI, 0 to 0) in 2019 (Fig. 2b, Table 1). The ASDR in Mongolia initially showed an increasing trend from 1990 to 1995 and then decreased to 2 (95% UI, 1 to 3) per 100,000 person-years in 2019. In China, the ASDR also showed a decline from 2 (95% UI, 1 to 2) to 0 (95% UI, 0 to 0) per 100,000 person-years from 1990 to 2019 (Fig. 2b, Table 1). In Japan and Singapore, the ASDR per 100,000 person-years declined prominently from 0 (95% UI, 0 to 0) in 1990 to 0 (95% UI, 0 to 0) in 2019 (Fig. 2b, Table 1).

### Time trends in the incidence of and death due to tuberculosis among adolescents and young adults aged 10–24 years from 1990 to 2019

**Fig. 3:**
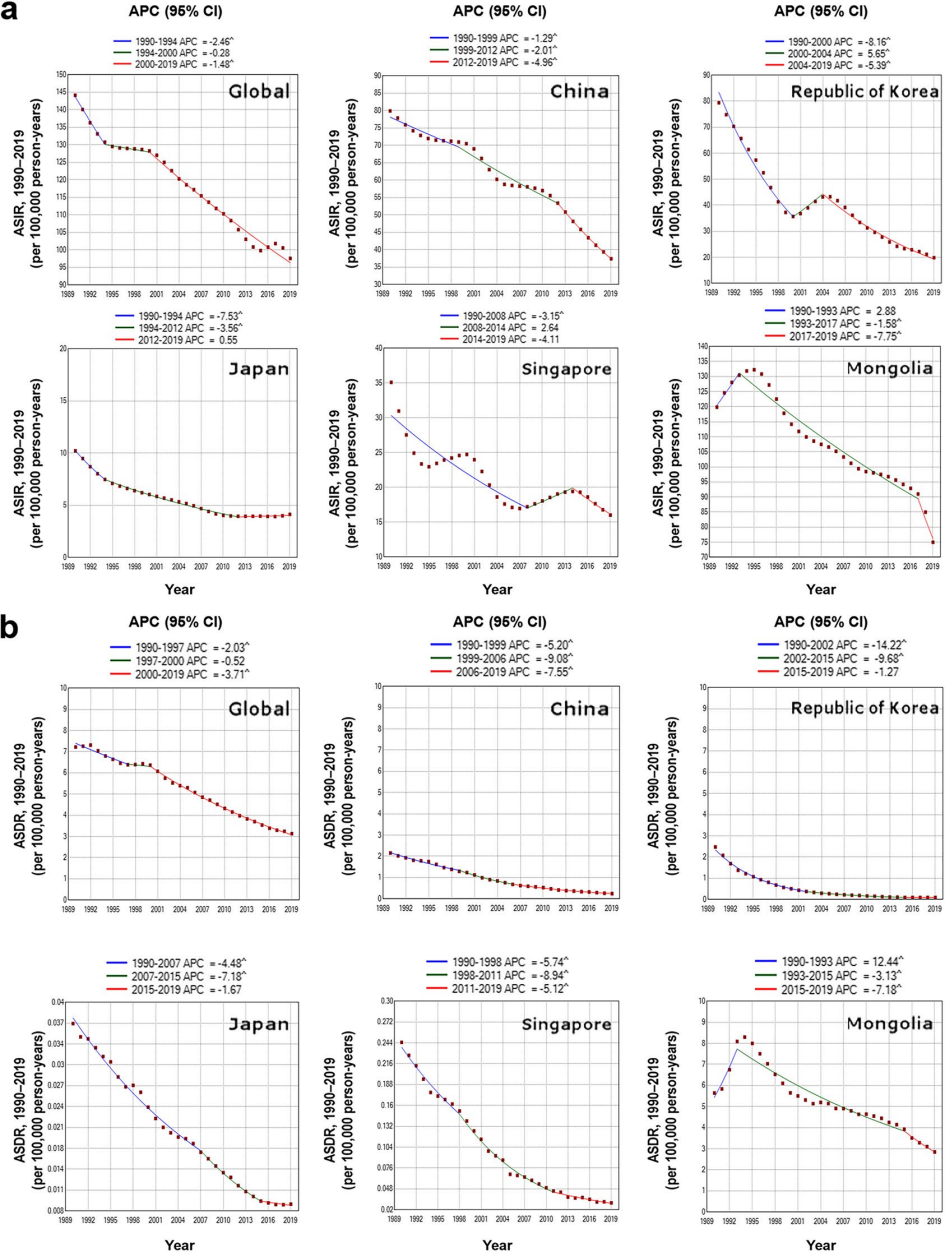
Estimates of APCs in the ASIRs (**a**) and ASDRs (**b**) of tuberculosis among adolescents and young adults aged 10–24 years worldwide and in five Asian countries from 1990 to 2019. ASDR, age-standardized death rate. ASIR, age-standardized incidence rate. APC, estimated annual percentage change. ^Trends between joinpoints were significantly different from zero (*P* < 0.05)

The greatest decreasing trend in the ASIR worldwide (APC, − 2.46) occurred from 1990 to 1994 (Fig. 3a), and the greatest decreasing trend in the ASDR worldwide (APC, − 3.71) occurred from 2000 to 2019 (Fig. 3b). For the ASIR, the greatest increasing trends were found in the Republic of Korea from 2000 to 2004 (APC, 5.65), Mongolia from 1990 to 1993 (APC, 2.88), and Singapore from 2008 to 2014 (APC, 2.64) (Fig. 3a). The greatest decreasing trends of the ASIR were found in the Republic of Korea from 1990 to 2000 (APC, − 8.16), Mongolia from 2017 to 2019 (APC, − 7.75), and Japan from 1990 to 1994 (APC, − 7.53) (Fig. 3a).

For the ASDR, the greatest increasing trends were found in Mongolia from 1990 to 1993 (APC, 12.44) (Fig. 3b). The greatest decreasing trends of the ASDR were found in the Republic of Korea from 1990 to 2002 (APC, − 14.22), 2002 to 2015 (APC, − 9.68), China from 1999 to 2006 (APC, − 9.08), and Singapore from 1998 to 2011 (APC, − 8.94) (Fig. 3b).

## Discussion

This study provides original estimates of the large burden of tuberculosis in adolescents and young adults aged 10–24 years global and in 5 Asian countries. We estimate that 1.82 million young people developed tuberculosis worldwide in 2019. There were significant decreases in the TB incidence rates and death rates worldwide and in 5 Asian countries from 1990 to 2019. These estimates were determined using the most comprehensive source of data extracted from the GBD 2019 [15]. TB in children and adolescents is an overlooked epidemic with a substantial disease burden [10]. It is estimated that nearly 1 million children develop TB each year [17], and there were an estimated 208,000 child deaths globally due to TB in 2019 [18]. Compared to early childhood, adolescence represents a period of increased susceptibility to TB, and the reasons for this are not completely understood, although it is thought that sex hormones, changing social contact patterns and immunological changes may each play a role [19]. According to a mathematical modelling study, an estimated 239,000 children younger than 15 years died from TB worldwide in 2015, and more than 70% of those deaths occurred in the southeast Asia and Africa regions [20]. Although adolescents and young adults (10–24 years) with TB do transmit TB and have present diagnostic and treatment challenges [21], to date, adolescents have not been addressed as a distinct population within TB control efforts, and we have scant knowledge of the burden and outcomes of TB in these age groups in Asian regions. Here, we carried out a comprehensive and systematic study to reveal the current burdens and trends in TB incidence and death in five Asian countries.

In this study, we mainly found that actions to control TB achieved positive results in both the age-standardized incidence rate and mortality from 1990 to 2019 among people aged 10–24 years worldwide and in five Asian countries for both sexes, especially in the Republic of Korea; however, TB mortality has shown a slow decline in recent years in the Republic of Korea and Japan (2015–2019). ASDR decreased significantly among those people in China (2006–2019) and Mongolia (2015–2019) in recent years. Notable downtrend differences were observed: the ASIR and ASDR steadily decreased in the Republic of Korea, China, Japan and Singapore from 1990 to 2019 but fluctuated in Mongolia, which initially showed an increasing trend from 1990 to 1995 and then decreased to 2 (95% UI, 1 to 3) per 100,000 person-years in 2019.

It was estimated that there would be 10.6 million new cases of TB worldwide in 2021, including 1.2 million children. We found that the ASIR and ASDR in China and Mongolia were higher than those in Japan, Singapore and the Republic of Korea. In recent years, economic levels were found to be related to TB mortality and incidence [22, 23], and it was predicted that malnutrition, smoking, alcohol abuse, human immunodeficiency virus infection, and diabetes contribute to higher TB morbidity and mortality [24]. Moreover, high economic levels in countries might be associated with less drug resistance [25]. In 2019, eight countries with a high TB burden accounted for two-thirds of the global total: six Asian countries, China was included (8.4%) [6]. The characteristics may be affected by the differences in socioeconomic determinants and health system development among these countries. TB prevention has been historically neglected in high TB burden settings due to resource constraints [26]. The Sustainable Development Goals have placed new emphasis on prevention, while standard practice in high-income countries and the updated WHO guidelines on treatment for M.tb infection include substantial expansion of the current policies in low- and middle-income countries, with direct relevance to adolescents.

The greatest increasing trends of the ASIR were found in the Republic of Korea from 2000 to 2004 and Mongolia from 1990 to 1993, and astonishingly, the greatest decreasing trends of the ASIR were found in the Republic of Korea from 1990 to 2000 and Mongolia from 2017 to 2019. These findings reflect the large differences in TB prevention and management among the five Asian countries.

There were certain reasons for the fluctuating changes in the ASIR and ASDR among them in the Republic of Korea from 1990 to 2019. In 2000, to assess the frequency and distribution of extensively drug-resistant (XDR) TB cases and to ensure the second-line drugs (SLDs) proper use to prevent increased drug resistance, World Health Organization (WHO) conducted a survey during 2000–2004, data on drug susceptibility of TB isolates were obtained from Republic of Korea, where 15% MDR TB cases were XDR [27]. Meanwhile, the diagnostic accuracy of the data reported in the Korean tuberculosis surveillance system (KTBS) has been doubted in 2012 [28]. They reviewed the clinical data of pulmonary tuberculosis (PTB) cases notified through KTBS in 2004. It conclued that where infection was not confirmed bacteriologically or histologically, only 60% of the cases were definite, probable, or possible. Only more than 70% of PTB notified in Korea can be regarded as real TB. Those above may be the reasons why The ASIR initially decreased from 1990 to 2000, then increased and peaked in 2005. After that, the government has increased budgets and strengthened patient management policies since 2011. The management of latent TB was added to the response with strengthened and extensive contact investigations in the five-year TB control plan (2013–2017), and implementation was established in 2013. Due to these efforts, Korea achieved an average 5.2% reduction annually in the TB incidence rate between 2011 and 2016. To further reduce the TB burden, the government has introduced additional measures, including mandatory latent TB infection screening for community workers in congregate settings, including daycare centers for children, kindergarten, and teachers in schools.

Mongolia was listed among the 30 countries with a high TB burden in 2021. Approximately 10–11% of the TB infections are occur in children, which is higher than the global average (6.0%) [29]. An earlier study showed that the lowest incidence of TB in the past 10 years occurred from 1990 to 1994, with a marked increase by 1995 in the whole population according to national statistics [30], consistent with our results. Unlike the continuing increasing trend of incidence through 1999 in the whole population, the ASIR among adolescents decreased through 2019 in our study. The increase probably represents a reporting bias resulting from a relative lack of evaluation available during the first few years after Mongolia was no longer under Soviet control and until the national TB program was established in 1994 [31].

In the 1990s, China increased BCG vaccination coverage to 90% and optimized vaccination methods under the guidance of the National Plan for Tuberculosis Prevention and Control (1990–2000) [32]. Since 1992, the World Bank has provided loans to support China in implementing DOTS in 13 provinces as a pilot program and then extending it to the whole country. The withdrawal of development assistance reduced China’s total spending on TB from 2000 to 2017, but government spending on TB increased by 4.7% per year [33]. Consistent political will, periodic epidemic surveillance and standard treatment processes greatly contributed to the continuous reduction of TB incidence and mortality in China.

In April 2005, the Tuberculosis Control Law was revised in Japan, which has newly emphasized medical screening. In March 2006, the draft for the revised Infectious Diseases Law was approved by the Cabinet of government, and the combination of the two laws indeed improved the program quality for the control of TB and infectious diseases [34]. A nationwide computerized TB surveillance system was revised in 2007, which ensured the planning and execution of TB control with the provision of useful epidemiological information from the system.

Following a series of defined environmental, housing and medical reforms, the disease rates in Singapore declined sharply from the 1960s [35], which resulted in a de-emphasis on TB relative to other diseases. Singapore did not lose sight of the illness. Government officials remained concerned about the prevalence of TB cases among some social groups, particularly elderly individuals, as there were patients who did not complete their treatment.

Smoking and alcohol use have historically been more common among males than females, which may help to explain the greater decrease in the ASIR and ASDR among females. Many adolescents experiment with alcohol and tobacco, both of which exacerbate TB risk and lead to worse outcomes [36]. The association between alcohol use and TB has long been known, and in vivo and in vitro studies have demonstrated that alcohol use significantly disrupts the immune response, increasing susceptibility to respiratory diseases such as TB [37].

In conclusion, there were significant decreases in the TB incidence rates and death rates among adolescents and young adults aged 10–24 years worldwide and in 5 Asian countries from 1990 to 2019, and the trends were especially significant in developed countries and varied across geographic regions. Beyond the health benefits associated with general improvements in socioeconomic status, specific local strategies are needed to further reduce the number of cases and deaths due to TB, such as revising public health measures and improving the life standards of adolescents and young adults in these regions. However, our study had some limitations. First, deficiencies in the databases included possible errors and potential biases during entry data, and data missing. Further studies of the epidemiology of tuberculosis among young people are needed, and for continued strengthening of tuberculosis surveillance systems and follow-up of latent tuberculosis infection in schools or colleges in Asian. Another limitation of the study is failure to examine the effect of related factors on the TB incidence such as individual’s socioeconomic status, nutritional factors, sexual development, and co-infection. Additional nation-wide and prospective studies in Asian are needed to better understand the characteristics of such patients. However, this work provides an important platform for increasing attention to this neglected group, who is very crucial in ending the global tuberculosis epidemic.

## Data Availability

This declaration is not applicable.

## References

[1] Sawyer SM, Afifi RA, Bearinger LH (2012). Adolescence: a foundation for future health. Lancet.

[2] Patton GC, Sawyer SM, Santelli JS (2016). Our future: a Lancet commission on adolescent health and wellbeing. Lancet.

[3] Morabia A (2014). Snippets from the past: cohort analysis of disease rates-another piece in a seemingly still incomplete puzzle. AM J EPIDEMIOL.

[4] Chenciner L, Annerstedt KS, Pescarini JM (2021). Social and health factors associated with unfavourable treatment outcome in adolescents and young adults with tuberculosis in Brazil: a national retrospective cohort study. LANCET GLOB HEALTH.

[5] Graham SM, Grzemska M, Brands A (2015). Regional initiatives to address the challenges of tuberculosis in children: perspectives from the Asia-Pacific region. INT J INFECT DIS.

[6] Chakaya J, Khan M, Ntoumi F (2021). Global Tuberculosis Report 2020 - Reflections on the Global TB burden, treatment and prevention efforts. Int J Infect Dis.

[7] Murray CJ, Ortblad KF, Guinovart C (2014). Global, regional, and national incidence and mortality for HIV, tuberculosis, and malaria during 1990–2013: a systematic analysis for the Global Burden of Disease Study 2013. Lancet.

[8] Lonnroth K, Jaramillo E, Williams BG (2009). Drivers of tuberculosis epidemics: the role of risk factors and social determinants. Soc Sci Med.

[9] Salehi M, Vahabi N, Pirhoseini H (2021). Trend analysis and longitudinal clustering of tuberculosis mortality in Asian and North African countries: results from the global burden of disease 2017 study. Med J Islam Repub Iran.

[10] Chiang SS, Dara M (2020). TB in children and adolescents. Int J Tuberc Lung Dis.

[11] Dodd PJ, Gardiner E, Coghlan R (2014). Burden of childhood tuberculosis in 22 high-burden countries: a mathematical modelling study. Lancet Glob Health.

[12] Kalra A (2017). Care seeking and treatment related delay among childhood tuberculosis patients in Delhi. India Int J Tuberc Lung Dis.

[13] Global burden of 369 diseases and injuries in 204 countries and territories, 1990–2019: a systematic analysis for the Global Burden of Disease Study 2019. Lancet. 2020;396:1204–1222. 10.1016/S0140-6736(20)30925-9 PMC7567026 33069326

[14] Global burden of 87 risk factors in 204 countries and territories, 1990–2019: a systematic analysis for the Global Burden of Disease Study 2019. Lancet. 2020;396:1223–1249. 10.1016/S0140-6736(20)30752-2 PMC7566194 33069327

[15] GBD 2019 Demographics Collaborators (2020). Global age-sex-specific fertility, mortality, healthy life expectancy (HALE), and population estimates in 204 countries and territories, 1950–2019: a comprehensive demographic analysis for the Global Burden of Disease Study 2019. Lancet.

[16] Kim HJ, Fay MP, Feuer EJ (2000). Permutation tests for joinpoint regression with applications to cancer rates. Stat Med.

[17] Marais BJ, Amanullah F, Gupta A (2020). Tuberculosis in children, adolescents, and women. Lancet Respir Med.

[18] Bagcchi S (2023). WHO's Global Tuberculosis Report 2022. Lancet Microbe.

[19] Snow KJ, Cruz AT, Seddon JA (2020). Adolescent tuberculosis. *Lancet Child **Adolesc*. Health.

[20] Dodd PJ, Yuen CM, Sismanidis C (2017). The global burden of tuberculosis mortality in children: a mathematical modelling study. Lancet Glob Health.

[21] Graham SM, Marais BJ, Amanullah F. Tuberculosis in Children and Adolescents: Progress and Perseverance. Pathogens. 2022;11. 10.3390/pathogens11040392 PMC9029126 35456067

[22] Reeves A, Basu S, McKee M (2015). Tuberculosis control and economic recession: longitudinal study of data from 21 European countries, 1991–2012. Bull World Health Organ.

[23] Alba S, Rood E, Bakker MI (2018). Development and validation of a predictive ecological model for TB prevalence. Int J Epidemiol.

[24] Tellez-Navarrete NA, Ramon-Luing LA, Munoz-Torrico M (2021). Malnutrition and tuberculosis: the gap between basic research and clinical trials. J Infect Dev Ctries.

[25] Song WM, Li YF, Liu YX (2021). Drug-Resistant Tuberculosis Among Children: A Systematic Review and Meta-Analysis. Front Public Health.

[26] Chang SH, Eitzman SR, Nahid P (2014). Factors associated with failure to complete isoniazid therapy for latent tuberculosis infection in children and adolescents. J Infect Public Health.

[27] Emergence of Mycobacterium tuberculosis with extensive resistance to second-line drugs--worldwide, 2000–2004. MMWR Morb Mortal Wkly Rep. 2006;55:301–305. 16557213

[28] Jeong I, Kim HJ, Kim J (2012). Diagnostic accuracy of notified cases as pulmonary tuberculosis in private sectors of Korea. J Korean Med Sci.

[29] Zanaa A, Paramita SA, Erdenee O (2022). Childhood Tuberculosis in Mongolia: Trends and Estimates, 2010–2030. Tohoku J Exp Med.

[30] Ebright JR, Altantsetseg T, Oyungerel R (2003). Emerging infectious diseases in Mongolia. Emerg Infect Dis.

[31] Toyota M (1998). Time trend in incidence and mortality of tuberculosis and characteristics of notified tuberculosis patients in urban area of Mongolia. Kekkaku.

[32] Yang WZ (2019). Dramatic achievements in infectious disease prevention and treatment in China during the past 70 years. Zhonghua Liu Xing Bing Xue Za Zhi.

[33] Su Y, Garcia BI, Harle AC (2020). Tracking total spending on tuberculosis by source and function in 135 low-income and middle-income countries, 2000–17: a financial modelling study. Lancet Infect Dis.

[34] Tsukahara T (2006). Tuberculosis Control Law of Japan: current issues and prospects of tuberculosis control plan. Kekkaku.

[35] Loh KS, Hsu LY (2019). Tuberculosis in Singapore: Past and Future. Ann Acad Med Singap.

[36] Francisco J, Oliveira O, Felgueiras O et al. How much is too much alcohol in tuberculosis? Eur Respir J. 2017;49. 10.1183/13993003.01468-2016 28100547

[37] Molina PE, Happel KI, Zhang P (2010). Focus on: Alcohol and the immune system. Alcohol Res Health.

